# Vogt-Koyanagi-Harada disease during prolonged intermittent steroid therapy for chronic obstructive pulmonary disease: a case report

**DOI:** 10.11604/pamj.2021.39.101.29904

**Published:** 2021-06-03

**Authors:** Shalini Akulwar, Hemant Gupta, Manas Pustake, Tushar Vidhale, Sumeet Lahane, Prafulla Jaya Rohan

**Affiliations:** 1 Department of Internal Medicine, Grant Government Medical College and Sir Jamshedjee Jeejeebhoy Group of Hospitals, Mumbai, India,; 2 Department of Ophthalmology, Grant Government Medical College and Sir Jamshedjee Jeejeebhoy Group of Hospitals, Mumbai, India,; 3 Department of Radiology, BGS Global Institute of Medical Sciences, Bangalore, India

**Keywords:** Vogt-Koyanagi-Harada disease, panuveitis, autoimmune disease, granulomatous autoimmune disease, case report

## Abstract

Vogt-Koyanagi-Harada Disease (VKHD) is a rare systemic granulomatous autoimmune condition that affects melanocyte-rich organs including the eyes, inner ears, meninges, skin, and hair. VKHD causes chronic uveal inflammation and a loss in visual acuity in some patients. Patients generally respond well to steroid therapy. In our patient, we evidenced VKHD in the chronic recurrent stage at the time of presentation while the patient was on intermittent systemic steroid therapy. To date, no cases of VKHD have been reported in patients who were taking immunosuppressive medications. This study sheds light on the possibility that, in addition to the complex multisystem autoimmune phenomenon, other variable factors may also be implicated in the etiopathogenesis of this disease. Also, if a patient presents with subacute vision loss and an acute onset headache and encephalopathy, this differential diagnosis should be kept in mind, and the patient should be treated as soon as possible if the diagnosis is confirmed.

## Introduction

Vogt-Koyanagi-Harada disease (VKHD) is a multisystemic autoimmune condition in which the organs with high melanin concentration, like the eyes, meninges, ears, and skin, are the primary targets of autoimmunity. This disorder typically manifests as bilateral granulomatous pan-uveitis with neurological, audiological, and integumentary features. The disease mostly affects genetically susceptible individuals and is triggered by environmental factors, including viral infections. Recent studies suggest that immunogenetics, rather than skin pigmentation, contributes more to disease predisposition [1-3]. We report a case of VKHD when the patient was being treated for chronic obstructive pulmonary disease (COPD) with intermittent oral steroid therapy.

## Patient and observation

**Figure 1:**
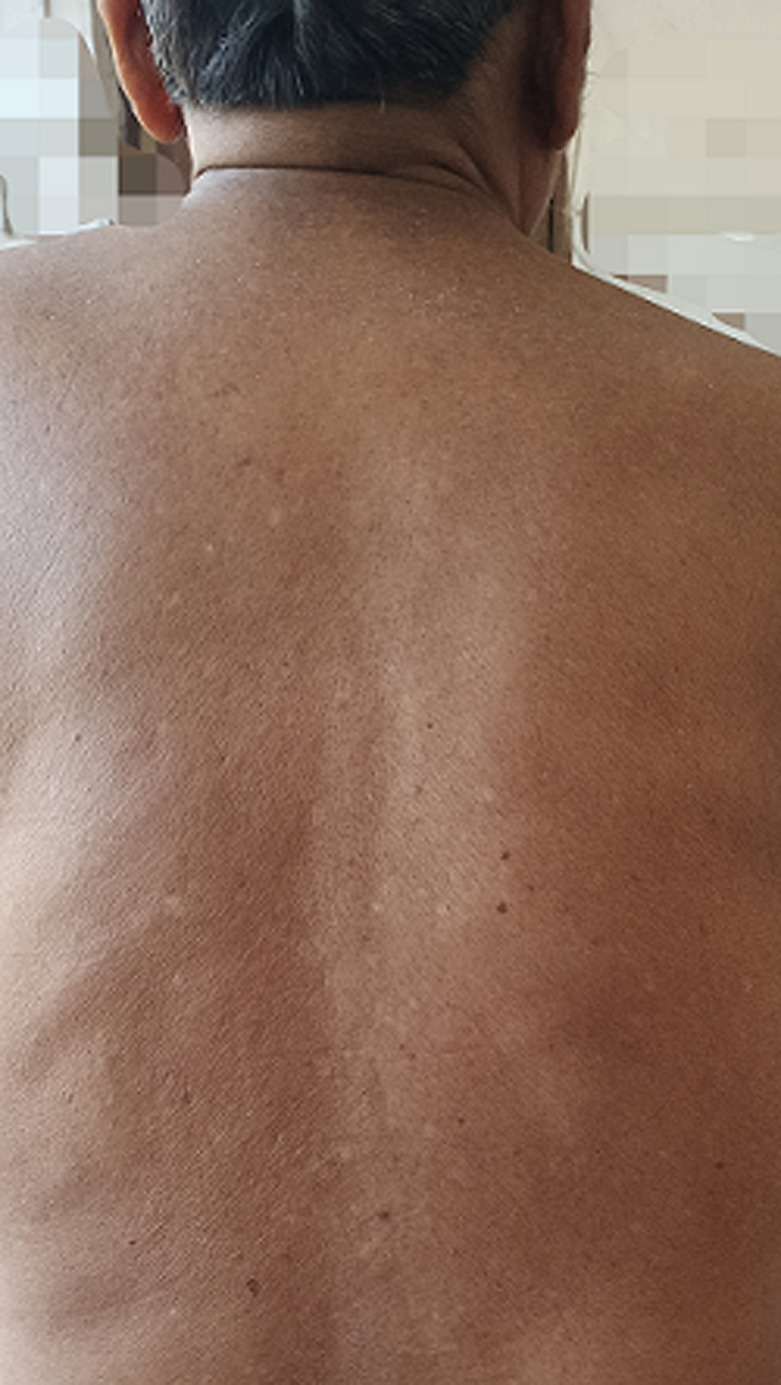
hypopigmented macules on back and trunk

**Figure 2:**
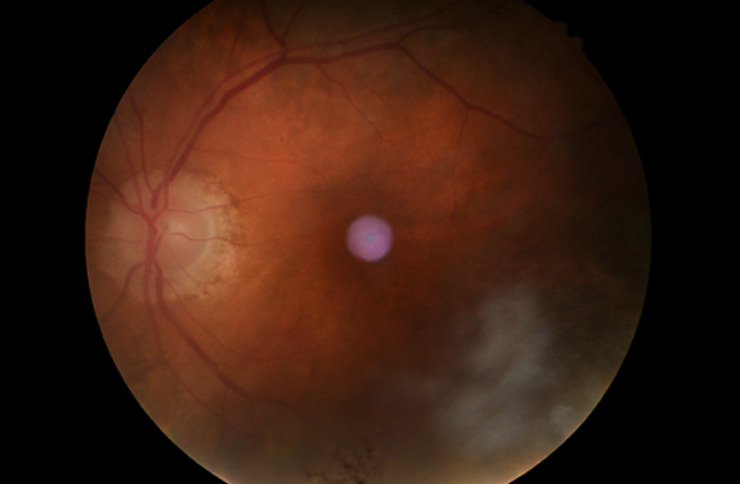
sunset glow fundus

A 68-year-old gentleman, presented to us with a history of blurring of vision for 45 days, severe holocranial headache with mild grade of evening rise in temperature for eight days, and acute loss of hearing in both ears for two days. The illness was acute in onset and progressive in nature. He had a severe holocranial headache, drowsiness and clouded sensorium at the time of presentation. He was a one-eyed patient with a history of loss of vision in the right eye status post-cataract surgery 28 years back. This time, he presented with blurring followed by a diminution of the vision of the left eye for more than a month, with associated redness and watering of the eyes. There was no history of any injury to the eye. He was an old case of COPD, poorly managed for ten years, and was repeatedly prescribed oral prednisolone for acute exacerbation of the same for the last six years. While taking the history of medications, he mentioned using oral steroids “as and when needed”. The patient was compliant to his inhaled steroid medications and his last oral prednisolone consumption was six months prior to the onset of ophthalmological symptoms. He had a history of primary pulmonary tuberculosis (TB) and was treated 20 years back with a full course of anti-tubercular medications. On examination, the patient was acutely ill, delirious, and was not oriented to time, location, or person; but could execute basic commands with considerable psychomotor slowing. His vitals were within normal limits, and all his reflexes were normal. Other than hearing loss and visual deterioration, there were no focal neurological deficits. He had nuchal rigidity. He also had several hypo-pigmented macules on his back and trunk (Figure 1). Since the patient had redness and lacrimation in both eyes, ophthalmological consultation was sought. His best-corrected visual acuity on ophthalmological examination was no perception of light in the right eye and finger counting close to the face in the left eye. The right eye had a complete leucomatous corneal opacity, obscuring any view of the anterior segment, and the ultrasound revealed retinal detachment with diffuse choroid thickening. The left eye revealed a non-reacting semi-dilated pupil, a normal anterior chamber, and old keratic precipitates on the endothelium. The retinal examination of the left eye revealed a pale optic disc with peripapillary atrophy and depigmented choroid (due to chronic disease), giving the impression of a “sunset glow fundus” (white optic disc surrounded by orange-red colored choroid) (Figure 2).

**Figure 3:**
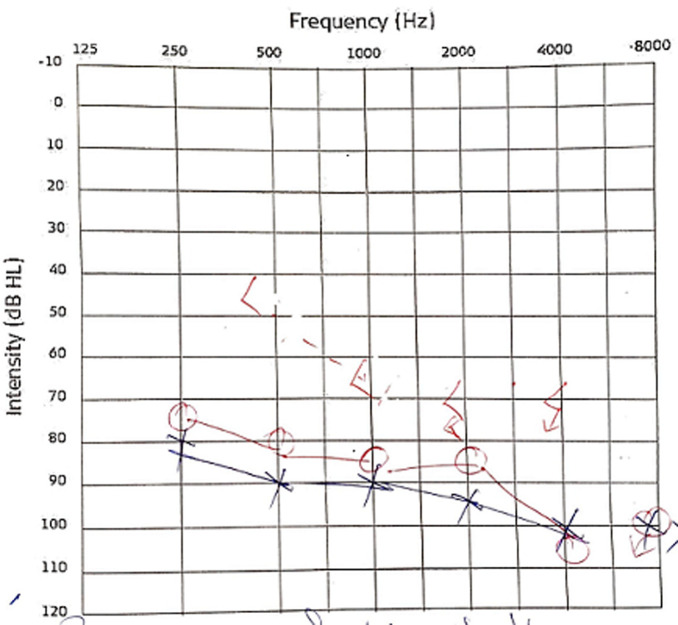
audiogram showing bilateral sensorineural hearing loss

Cerebrospinal fluid (CSF) examination revealed lymphocyte-predominant pleocytosis and raised protein (63 mg/dl). Cerebrospinal fluid adenosine deaminase (ADA) was 3.30 units/L (normal range 0-9 units/L). High-resolution computed tomography (HRCT) chest revealed bilateral para-septal and centrilobular emphysema with bullae formation predominantly in bilateral upper lobes. His spirometry was suggestive of an obstructive pattern. His magnetic resonance imaging (MRI) brain revealed multiple lacunar infarcts. His pure tone audiometry showed bilateral sensorineural hearing loss ranging from severe to profound (Figure 3). Due to acute encephalopathy, nuchal rigidity with CSF pleocytosis, diagnosis of meningoencephalitis was considered. However, when the ophthalmologic examination findings were taken into account, the granulomatous uveitis along with meningoencephalitis, auditory (sensory neural hearing loss), and skin involvement (vitiligo), rather than isolated meningitis, were strongly pointing towards VKHD. Other differentials were ruled out using proper investigations. Considering the imminent possibility of sight-threatening complications, the patient was immediately initiated on intravenous methylprednisolone, followed by oral tablet prednisolone with the plan for slow tapering in the next six months. He also received supportive management, like topical local application of tacrolimus, 1% ointment for hypo-pigmented patches, oral calcium, and vitamin D supplements. The patient responded to corticosteroids dramatically. His sensorium improved after three days of treatment; he became alert, could execute complex commands, and was oriented to time, place, and person. After one week, his left eye visual acuity improved to 6/36, and he reported subjective hearing improvement after two weeks. Even so, after two months of therapy, repeated pure tone audiometry revealed no objective signs of improvement. The patient was informed of the recurring nature and poor progression of the disease due to chronicity.

## Discussion

In VKHD, the diagnosis is largely clinical. Asians and women between the ages of 20-50 and people expressing HLA DRB1*0405 are primarily at risk [1-4]. It evolves through four stages - prodromal, acute uveitic, convalescent, and chronic recurrent. However, not every patient will go through all these stages [1-3]. Our patient initially had redness, lacrimation, and blurring of eyes because of panuveitis which progressed over a month, and at the presentation, he already had choroidal depigmentation which suggests the disease to be in the chronic recurrent phase. The immunosuppressive action of steroids is well-known. The patient was on high doses of oral and inhaled steroids for controlling his COPD. Despite the patient's repeated use of steroids over five years, an autoimmune disorder developed. Etiologically, VKHD is believed to be caused by complex autoimmune responses against melanocytes (primarily of CD4+ T helper cells). This evidently indicates that other causes, rather than a single one (autoimmune phenomenon), are playing a significant role in the pathogenesis of this disorder. It may also be a possibility that intermittent dosages of over-the-counter steroids disguised his underlying VKHD for long time. As a result, he had an exacerbation in the form of acute meningoenchephalitis over the chronic underlying disease. Studies are needed to further unveil the hidden complex mechanisms of pathogenesis of the disease. Initiation of high-dose corticosteroids (pulse treatment with intravenous methylprednisolone 1gm/day for 3 days or oral prednisolone 1-2 mg/kg/day), followed by oral prednisolone 1mg/kg/day for at least 4 months with slow tapering during the next 4 to 6 months or more is the key pillar of therapy for VKHD [1-3, 5,6]. Patients, in general, respond well to this care, as did our patient.

## Conclusion

A patient presenting with a history of subacute diminution of vision (due to chronic uveitis) with superadded acute onset of headache and encephalopathy (due to acute meningoencephalitis) should be suspected for VKHD. Moreover, uveitis is associated with serous retinal detachment is a dead giveaway for VKHD. Development of the disease during steroid therapy suggests that, in addition to the complex multisystem autoimmune phenomenon, other variable factors may also be implicated in the etiopathogenesis of this disease. While it is an unusual condition, given the possibility of a dramatic and complete response with early aggressive immunosuppressive therapy, this illness should be included in the differential diagnosis of recurrent aseptic meningitis with ocular disease.
